# Cardiomyocyte Contractility and Autophagy in a Premature Senescence Model of Cardiac Aging

**DOI:** 10.1155/2020/8141307

**Published:** 2020-04-14

**Authors:** Steffen Häseli, Stefanie Deubel, Tobias Jung, Tilman Grune, Christiane Ott

**Affiliations:** ^1^Department of Molecular Toxicology, German Institute of Human Nutrition Potsdam-Rehbrücke (DIfE), Nuthetal 14558, Germany; ^2^German Center for Cardiovascular Research (DZHK), Partner Site Berlin 13357, Germany; ^3^German Center for Diabetes Research (DZD), Munich-Neuherberg 85764, Germany; ^4^NutriAct-Competence Cluster Nutrition Research Berlin-Potsdam, Nuthetal 14558, Germany; ^5^University of Potsdam, Institute of Nutrition, Nuthetal 14588, Germany

## Abstract

Globally, cardiovascular diseases are the leading cause of death in the aging population. While the clinical pathology of the aging heart is thoroughly characterized, underlying molecular mechanisms are still insufficiently clarified. The aim of the present study was to establish an *in vitro* model system of cardiomyocyte premature senescence, culturing heart muscle cells derived from neonatal C57Bl/6J mice for 21 days. Premature senescence of neonatal cardiac myocytes was induced by prolonged culture time in an oxygen-rich postnatal environment. Age-related changes in cellular function were determined by senescence-associated *β*-galactosidase activity, increasing presence of cell cycle regulators, such as p16, p53, and p21, accumulation of protein aggregates, and restricted proteolysis in terms of decreasing (macro-)autophagy. Furthermore, the culture system was functionally characterized for alterations in cell morphology and contractility. An increase in cellular size associated with induced expression of atrial natriuretic peptides demonstrated a stress-induced hypertrophic phenotype in neonatal cardiomyocytes. Using the recently developed analytical software tool *Myocyter*, we were able to show a spatiotemporal constraint in spontaneous contraction behavior during cultivation. Within the present study, the 21-day culture of neonatal cardiomyocytes was defined as a functional model system of premature cardiac senescence to study age-related changes in cardiomyocyte contractility and autophagy.

## 1. Introduction

Aging is a time-dependent process with a progressive reduction in the physiological and functional capacity as well as in stress resilience [1]. In 2016, with 44%, cardiovascular diseases accounted for the main cause of death from noncommunicable diseases worldwide, whereas an age dependency of mortality with a rapid onset from 60 years of age was shown [2–4].

The phenotype of the aging heart is characterized by a gradual loss of cardiac function [5]. With higher age, a primary impairment of diastolic function emerges, which under increasing workload expands to a reduction of heart rate and systolic ejection capacity [6]. Acute hemodynamic stress can be compensated by (neuro-)hormonal systems and physiological hypertrophy [6]. According to this, the cardiac-derived hormones atrial natriuretic peptide (ANP) and brain natriuretic peptide (BNP) fulfill a vasodilatory, natriuretic, and diuretic function [7]. However, the chronic demand of compensatory mechanisms leads to a pathophysiological state of the heart [8]. The excessive secretion of ANP and BNP serves as a clinical marker of ventricular hypertrophy, hypertension, heart failure, and myocardial infarction [7].

Functional and structural changes of the aging heart are directly linked to an impairment of cardiac myocytes [9]. On a cellular level, cardiac decline is associated with a dysregulation of Ca^2+^ homeostasis and reorganization of the contractile apparatus, a dysfunction of mitochondria, rise of oxidative stress and accumulation of misfolded proteins, increase in cell size, and apoptotic as well as necrotic cell death [6, 9]. Briefly after birth, the majority of mammalian cardiomyocytes enter a postmitotic state of terminal differentiation and efficient tissue regeneration is lost [10, 11]. The restricted proliferation of cardiomyocytes prevents a mitotic dilution of damaged structures [9]. Therefore, the maintenance of cardiac homeostasis is highly dependent on cellular mechanisms of structural quality control [12, 13].

Cellular homeostasis involves a constitutive cycle of synthesis and degradation of proteins and organelles [14]. The evolutionary-conserved autophagy-lysosomal system (ALS) is responsible for the engulfment and successive catabolism of macromolecules, protein aggregates, and cell organelles up to the supply of degradation products to cellular metabolism [14, 15]. Induction of macroautophagy, hereafter referred to as autophagy, results in the recruitment of autophagy-related (ATG) proteins to characteristic membrane structures primarily located at the ER [15, 16]. The following formation of two regulatory complexes, surrounding unc-51 like autophagy activating kinase 1 (Ulk1) and class III phosphatidylinositol 3-kinase (PI3KC3), initiates the *de novo* synthesis of a membranous, cup-shaped structure, the so-called phagophore. Two ubiquitin-like systems are involved in the expansion of the isolation membrane. Firstly, ATG12 conjugates with ATG5 and the ATG12-ATG5 conjugate further establishes a complex with ATG16L. Then, nascent microtubule-associated protein 1 light chain 3 (LC3) is cleaved to LC3-I and ligated to phagophore-associated phosphatidylethanolamine in an ubiquitin-like manner to form the membrane-bound, ligated form LC3-II [16–18]. To enable selective degradation via the ALS, substrates are polyubiquitinated by linkage at position Lys^63^ of ubiquitin, recognized by the autophagic cargo receptor sequestosome 1 (p62), and transported to the isolation membrane in an interaction with LC3-II [19]. The phagophore closes to a double membranous vesicle, the autophagosome, where the outer membrane fuses with a lysosome to form the autolysosome, eventually degrading the luminal cargo [15, 17].

Both LC3 and p62 are degraded in the autolysosome [19] and thus may serve as an indirect measure of working autophagy [20, 21]. However, due to the dynamic nature of the ALS, quantification of LC3 and p62 at a given point in time does not indicate substrate turnover *per se*. To evaluate the autophagic flux, it is highly recommended to compare autophagy in basal conditions with an introduced state of blocked lysosomal degradation [22]. Inhibition of v-ATPase by, e.g., concanamycin A (ConA) prevents acidification of lysosomes and impairs luminal hydrolases, resulting in an accumulation of sequestered cargo, among others LC3-II and p62. The relation of LC3-II and p62 using an inhibitor in comparison without lysosomal blockade illustrates the occurring transport of substrates by autophagy into the autolysosomes and is commonly used to determine the autophagic flux [20, 22].

The regulation of the ALS is dependent on energy and nutrient status, growth factors, oxidative and proteotoxic stress, hypoxia, and mechanical load, mainly permitting a cytoprotective adaptation [14, 15]. The transcription factor EB (TFEB) is a positive regulator of lysosomal biogenesis and among others induces p62 and LC3 expression [23]. On a posttranslational level, two main regulators of cellular energy status, mechanistic target of rapamycin kinase (mTOR) and AMP-activated protein kinase (AMPK), play a superordinate role in autophagy regulation. Acting as molecular sensor of nutrient, energy, and redox homeostasis, under favoring conditions mTOR promotes cell growth by stimulating biosynthesis and inhibiting autophagy [24]. Under energy deficiency, AMPK activates catabolic systems, such as autophagy, and inhibits anabolic pathways [24].

Increasing evidence suggests a decline of ALS in the aging heart [12, 13]. Accordingly, inactivation of autophagy in the mouse heart through tissue-specific deletion of ATG5 resulted in premature onset of an age-dependent functional decline. The experimentally induced heart failure was accompanied by cardiac hypertrophy, contractile dysfunction, accumulation of protein aggregates, disorganization of sarcomeres, and loss of mitochondrial function [25, 26]. Also, an age-dependent imbalance of mTOR and AMPK signaling is associated with decreased cardiac stress resistance [27, 28].

As model systems for *in vitro* studies of human cardiomyocyte aging are still limited [29], animal models remain a crucial tool to gain knowledge of cardiac (patho-)physiology [30]. While differentiated stem cells (embryonic [31] or induced pluripotent [32]) and immortalized cell lines (*e.g.*, HL-1 [33] and AC16 [34]) offer alternative approaches to research on single cell level, primary isolated cardiomyocytes seem to show the greatest resemblance of *in vivo* structure and functionality [30, 35]. In contrast to their adult state, mammalian neonatal cardiomyocytes allow the maintenance of a prolonged, physiologically contractive culture [36]. Murine neonatal cardiomyocytes have already been used to mimic diverse states of cardiac dysfunction, such as myocardial ischemia [37], ventricular hypertrophy [38], arrhythmia [39], and cellular senescence [40]. As studies on protein homeostasis (proteostasis) and contractility in cardiomyocyte aging remain a challenging task, culture of neonatal cardiomyocytes offers an optimal approach for manipulation studies under controlled conditions.

The objective of the present study was to establish a functional model of cellular cardiac aging in a short time span. Therefore, primary cardiomyocytes from neonatal mice were cultured over the course of 21 days and characterized on biomarkers of cellular senescence, cardiac hypertrophy, contractility, and autophagy.

## 2. Materials and Methods

### 2.1. Experimental Model and Primary Cell Isolation

Experiments were performed in cardiac myocytes derived from neonatal C57Bl/6J mice (Jackson Laboratory) in the age of 1-3 days. Animal housing conditions and experimental procedures were performed according to the National Institutes of Health guidelines of German Law on the protection and use of laboratory animals. As animals were exclusively sacrificed to collect organs and tissues for scientific purposes, no further approval by the national ethics committee was needed (§7 Abs.2 TierSchG).

Isolation of primary cardiomyocytes was performed using the *Pierce™ Primary Cardiomyocyte Isolation Kit* (Thermo Fisher Scientific, Waltham, USA; #88281) according to the manufacturer's instructions. Neonatal mice were decapitated with surgical scissors, and the heart was excised via sternotomy. Using a sterile scalpel, freshly obtained cardiac tissue was minced and subsequently washed with the implied *Hanks' Balanced Salt Solution* (HBSS) before cardiomyocytes were isolated by enzymatic digestion. The cells were suspended in tempered (37°C) *Dulbecco's Modified Eagle Medium* (DMEM) supplemented with 10% heat-inactivated FBS (Merck, Darmstadt, Germany; #F2442) and 1% penicillin/streptomycin (Biochrom, Berlin, Germany; #A2212). The isolation procedure was completed within 1 h.

### 2.2. Cardiomyocyte Culture

Culture dishes were precoated with 0.5% (*v*/*v*) fibronectin (Merck; #F1141) in 0.02% (*w*/*v*) gelatin (Merck; #G9391) solution for 1 h at 37°C and washed once with PBS before usage. Neonatal cardiomyocytes were cultured in supplemented DMEM at 37°C, 5% CO_2_, and 95% humidity atmosphere. After 24 h, culture medium was exchanged with fresh DMEM containing 1 *μ*l/ml growth supplement included in the isolation kit. Used culture medium was replaced with fresh DMEM without growth supplement on days 3, 6, 9, 13, and 17 post primary cell isolation. Neonatal cardiomyocytes were cultured for a period of 21 days in total.

### 2.3. Measurement of Cellular Contractility

To characterize the contractile behavior of neonatal cardiomyocytes, the recently developed macro *Myocyter* (v. 1.0), an analytical software tool for the image processing software *ImageJ* (v. 1.52b), was used [41]. By scaling the time-dependent changes of pixel intensity in subsequent frames of recorded cardiomyocytes, *Myocyter* enables the depiction of cellular contractility as positive amplitudes on an arbitrary 8-bit scale from 0 to 255. The experimental set-up consisted of a commercially available smartphone (Apple, Cupertino, USA; iPhone 6S) connected to the ocular of a confocal laser scanning microscope (Carl Zeiss, Oberkochen, Germany; LSM780) via a camera adapter (Svbony, Hong Kong, China). Nonelectrically stimulated, spontaneous contractions of neonatal cardiomyocytes were recorded at 120 frames per second for 20-30 s at 400-fold magnification (objective LD Plan-Neofluar 40x/0.6 Korr M27) in the transmitted light modus. Data extraction with *Myocyter* was performed according to the developer's instructions [41].

### 2.4. Determination of Autofluorescence

By the specific selection of excitation and emission wavelengths, the intrinsic autofluorescence from endogenous fluorophores of biological systems may be adjusted to the detection of oxidized protein aggregates up to aging-related lipofuscin pigments [42, 43]. Autofluorescence of cultured neonatal cardiomyocytes was measured with a confocal laser scanning microscope [44]. Contracting cells were excited at a wavelength of 405 nm (laser intensity 4.0%), and emission light was captured in a range of 410-585 nm at 400-fold magnification. For in parallel captured, congruent bright field images, masks around the edges of cardiomyocytes were created using the software *Corel® Photo-Paint® X3* (Corel Corporation, Ottawa, Canada; v. 13.0.0.739). The generated masks were conferred on the fluorescent images, and the average intensity of autofluorescence per cell was determined.

### 2.5. Immunofluorescence Staining

For immunofluorescence staining, cardiomyocytes were cultured on fibronectin/gelatin-coated glass bottom dishes (MatTek Corporation, Ashland, USA). Cultured cells were washed with PBS and fixed for 6 min with diethyl ether/ethanol mixture (1 : 1) at -20°C. Fixed cardiomyocytes were washed with PBS and incubated with 1% FBS in PBS for 30 min. Primary antibodies were diluted in PBS, and cells were incubated for 2 h in a humidified chamber at room temperature. Goat anti-*α*-actinin (ACTN1) antibody (Novus Biologicals, Centennial, USA; #AF8279) and mouse anti-p21/CDKN1A antibody (Thermo Fisher Scientific; #AHZ0422) were used as primary antibodies. Afterwards, cardiomyocytes were washed with PBS and incubated with secondary antibodies conjugated to Alexa Fluor® 546 nm (Thermo Fisher Scientific; #A-11056) and 647 nm (Abcam, Cambridge, UK; #ab150107) for 30 min in a lightproof, humidified chamber at room temperature. Samples were covered with Roti®-Mount FluorCare including DAPI (Carl Roth, Karlsruhe, Germany; #HP20.1) as mounting medium.

Microscopic visualization was carried out using a confocal laser scanning microscope at 630-fold magnification (objective Plan-Apochromat 63x/1.40 Oil DIC M27). Cardiomyocytes were defined by their characteristic sarcomeric striations elucidated by ACTN1 staining. The cell area was calculated via *Zen 2012 SP5* (*black*), *LSM 780* (Carl Zeiss, Jena, Germany; v. 14.0.0.0). To determine p21 in neonatal cardiomyocytes, an overlap between DAPI and p21 stained nuclei in ACTN1-positive cells was analyzed using the software *FIJI* (v. 1.52n).

### 2.6. Senescence-Associated *β*-Galactosidase Staining

A cytochemical determination of senescence-associated *β*-galactosidase (SA-*β*-Gal) activity at pH 6 [45] was performed using the *Senescence β-Galactosidase Staining Kit* (Cell Signaling, Danvers, USA; #9860) according to the manufacturer's guidelines. Stained cardiomyocytes were analyzed qualitatively on a standard microscope (Olympus Corporation, Tokyo, Japan; inverse microscope IX53P1 F) as SA-*β*-Gal-positive cardiac myocytes per total number of heart muscle cells.

### 2.7. Real-Time PCR Analysis

To isolate mRNA from cultured neonatal cardiomyocytes, *Dynabeads*™ *mRNA DIRECT*™ *Kit* (Thermo Fisher Scientific; #61012) was used according to the supplier's protocol. Therefore, cell lysates were incubated with oligo (dT)_25_ conjugated magnetic beads and hybridized mRNA was subtracted with a magnet. Subsequent to extraction, cDNA synthesis was performed using the *SensiFAST*™ *cDNA Synthesis Kit* (Bioline Reagents, London, UK; #BIO-65054) according to manufacturer's instructions, and samples were diluted 1 : 10 in nuclease-free water (Carl Roth; #T143). With a final volume of 25 *μ*l per reaction, mixtures for real-time PCR (qPCR) analyses contained 2.5 *μ*l 10x *DreamTaq™ Buffer* and 0.13 *μ*l *DreamTaq™ Hot Start DNA-Polymerase* (Thermo Fisher Scientific; #EP1702), 1 *μ*l cDNA template, 2 mM dNTPs (Bioline; #BIO-39028), 1x SYBR™ Green I (Thermo Fisher Scientific; #S7563), and 1 *μ*M forward and reverse primer. Murine primers were designed for the quantification of ANP (forward: 5′-AGGAGAAGATGCCGGTAGAAGA-3′, reverse: 5′-GCTTCCTCAGTCTGCTCACTCA-3′), BNP (forward: 5′-CACCGCTGGGAGGTCACT-3′, reverse: 5′-GTGAGGCCTTGGTCCTTCAA-3′), marker of proliferation Ki-67 (forward: 5′-AATCCAACTCAAGTAAACGGGG-3′, reverse: 5′-TTGGCTTGCTTCCATCCTCA-3′), LC3 (forward: 5′-GACCAGCACCCCAGTAAGAT-3′, reverse: 5′-T GGGACCAGAAACTTGGTCT-3′), p16/CDKN2A (forward: 5′-GAACTGCGAGGACCCCACTACC-3′, reverse: 5′-CAGGCATCGCGCACATCCA-3′), p62 (forward: 5′-AGATGCCAGAATCGGAAGGG-3′, reverse: 5′-GAGAGGGACTCAATCAGCCG-3′), proliferating cell nuclear antigen (PCNA) (forward: 5′-AGAGGAGGCGGTAACCATAGAG-3′, reverse: 5′-ACTGTAGGAGACAGTGGAGTGG-3′), and TFEB (forward: 5′-AGGAGCTGGGAATGCTGAT-3′, reverse: 5′-CTTGAGGATGGTGCCTTTGT-3′) and obtained from Sigma-Aldrich (St. Louis, USA) or BioTeZ (Berlin, Germany). Cycle conditions consisted of an initial heat activation at 95°C for 3 min followed by 40 cycles of denaturation at 95°C for 15 s, primer hybridization at 60°C for 30 s, and elongation at 72°C for 30 s. Product specificity was monitored via melting curve analysis. The relative mRNA expression levels of target genes were quantified via standard curves of amplified primer-specific cDNA with the *MxPro qPCR Software* (Agilent Technologies, Santa Clara, USA; v. 4.10). With the amplification of *β*-Actin (forward: 5′-CACTGCCGCATCCTCTTCCT-3′, reverse: 5′-GATTCCATACCCAAGAAGGAAGGC-3′), glyceraldehyde-3-phosphate dehydrogenase (GAPDH) (forward: 5′-GGGTGTGAACCACGAGAAAT-3′, reverse: 5′-GTCTTCTGGGTGGCAGTGAT-3′), hypoxanthine phosphoribosyltransferase 1 (HPRT) (forward: 5′-GCAGTCCCAGCGTCGTG-3′, reverse: 5′-GGCCTCCCATCTCCTTCAT-3′), and ribosomal protein L13a (RPL13a) (forward: 5′-GTTCGGCTGAAGCCTACCAG-3′, reverse: 5′-TTCCGTAACCTCAAGATCTGCT-3′), a normalization factor was calculated and used as internal standard [46].

### 2.8. Immunoblot Analysis

Proteins were either (i) collected in parallel to mRNA isolation as eluates after the binding of hybridized mRNA to the magnet, (ii) obtained as secretory proteins from the culture medium at days 6, 9, 13, 17, and 21 post primary cell isolation, or (iii) by direct uptake of cultured neonatal cardiomyocytes in reducing Laemmli sample buffer (0.25 mM Tris (pH 6.8), 40% Glycerol, 20% 2-Mercaptoethanol, 8% SDS, 0.03% Bromophenol Blue). To precipitate proteins from the supernatant collected during mRNA isolation and culture medium, the samples were incubated with 3x the volume of acetone (VWR, Radnor, USA; #20066) for 24 h at -20°C. Proteins were pelleted via centrifugation for 10 min at 25000 g and 4°C and acetone was discarded. The protein pellets were recovered in reducing sample buffer followed by denaturation for 5 min at 95°C.

Protein separation was conducted via SDS polyacrylamide gel electrophoresis on 7.5% and 15% polyacrylamide gels and with a standardized system (Biometra, Jena, Germany). Following gel electrophoresis, proteins were transferred and immobilized onto nitrocellulose membranes (Merck; Amersham™ Protran®) via a semidry blotting system (Bio-Rad, Hercules, USA). Membranes were blocked for 1 h at room temperature in blocking buffer (LI-COR, Lincoln, USA; #927-40000) diluted 1 : 5 in PBS. Primary antibodies were diluted in blocking solution with 0.1% Tween® 20 (Merck; #P9416) and incubated overnight at 4°C. Anti-AMPK subunit *α* antibody (Cell Signaling; #5832), anti- AMPK*α*_(Thr172)_ antibody (Cell Signaling; #2535), anti-ANP antibody (Novus Biologicals; #NBP2-14872), anti-GAPDH antibody (Abcam; #ab8245), anti-Lys^63^-linkage specific polyubiquitin antibody (Cell Signaling; #5621), anti-LC3 antibody (Cell Signaling; #12741), anti-mTOR antibody (Cell Signaling; #2983), anti-p53 antibody (Abcam; #ab131442), anti-SQSTM1/p62 antibody (Abcam; #ab56416), anti-p70 S6 kinase (p70S6k) antibody (Cell Signaling; #2708), and anti-p70S6k_(Thr389)_ antibody (Cell Signaling; #9234) were used as primary antibodies for immunoblot detection. Secondary antibodies conjugated to IRDye® 680LT (LI-COR; #926-68022) and 800CW (LI-COR; #926-32211) were diluted in blocking solution with 0.1% Tween® 20 and incubated in a lightproof cartridge for 1 h at room temperature. Membranes were scanned using the *Odyssey® CLx Imaging System* (LI-COR) and analyzed with the software *Image Studio™* (LI-COR; v. 4.0.21).

### 2.9. Coomassie Staining of Polyacrylamide Gels

Polyacrylamide gels were incubated with Coomassie staining solution (0.1% Coomassie® Brilliant Blue R 250, 42.5% Ethanol, 10% Acetic acid, 5% Methanol) for 10 min at room temperature which was exchanged for Coomassie destaining solution (10% Methanol, 7% Acetic acid) and incubated further for 16 h under slow seesawing motion. Stained gels were scanned using the *Odyssey® CLx Imaging System*.

### 2.10. Analysis of Autophagy Flux Using Concanamycin A

To evaluate the autophagic flux, neonatal cardiomyocytes were treated for 6 h with ConA, followed by protein analysis of autophagic proteins LC3 and p62 in comparison to the untreated control. A 1 mM DMSO-stock solution of ConA (Merck; #C9705) was diluted in culture medium to reach a final concentration of 2.5 nM (0.00025% DMSO).

### 2.11. Statistical Analysis

Experiments were conducted with at least 3 biological replicates, and results are presented as mean values ± SD. Statistical analysis was carried out using the software *GraphPad Prism* (GraphPad Software, San Diego, USA; v. 8.0.0). Differences between groups were assessed by two-tailed, unpaired *Student*'s *t*-test or one-way ANOVA followed by *Tukey*'s post hoc test. Statistical significance was considered and accordingly indicated at *p* < 0.05.

## 3. Results

**Figure 1:**
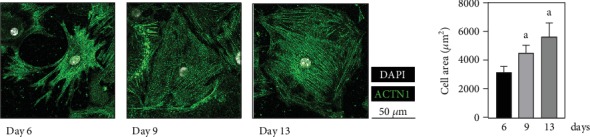
Cellular area of murine, neonatal cardiomyocytes during cultivation. Illustrated are representative images of neonatal cardiac myocytes immunofluorescently stained for ACTN1 (green) and DAPI (white) at indicated time points post primary cell isolation. Areas of cells with characteristic sarcomeric striations elucidated by ACTN1 were quantified (*n* = 4 mice). Data are presented as mean values ± SD. Statistical significance was assessed by one-way ANOVA (*p* < 0.05); ^a^reference day 6.

Murine, neonatal cardiomyocytes were cultured for a period of 21 days following primary cell isolation. After 24 h in culture, cardiomyocytes developed elongated, pseudopodial extensions and showed a contractile phenotype with typical spontaneous, concentric contractions. At 6 days post cell isolation, intercellular connections were observed which concluded in a synchronization of contractility. With a cell area of 3131 ± 421 *μ*m^2^ at day 6, a significant increase in cardiomyocyte size to 5659 ± 929 *μ*m^2^ at day 13 was detected (Figure 1). After 13 days, the cardiomyocyte culture was single-layered confluent. At the following time points, heart muscle cells were partly overgrown by ACNT1-negative noncardiomyocytes which prevented the explicit microscopic examination of ACTN1-positive cardiac myocytes.

**Figure 2:**
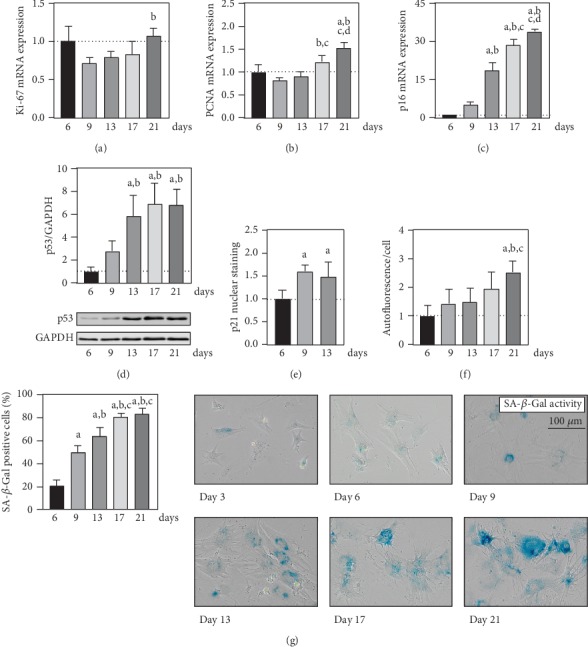
Cellular senescence in cultured, neonatal cardiomyocytes. Assessed were discriminative biomarkers of cellular senescence in murine cardiac myocytes at indicated time points post primary cell isolation. Relative mRNA expressions of (a) Ki-67, (b) PCNA, and (c) p16 were quantified via qPCR analyses (*n* = 4 mice). (d) Protein levels of p53 were determined by immunoblot analyses normalized to GAPDH, and representative blots are illustrated (*n* = 4 mice). (e) The signal of immunofluorescently stained p21 in cardiomyocyte nuclei and (f) autofluorescence per cardiac myocytes were microscopically quantified (*n* = 4 mice). (g) As validated biomarker of cellular senescence, the SA-*β*-Gal activity at pH 6 was measured qualitatively as positively stained cardiomyocytes per total number of heart muscle cells (*n* = 6 mice). Data are presented as mean values ± SD. Statistical significance was assessed by one-way ANOVA (*p* < 0.05); ^a^reference day 6; ^b^reference day 9; ^c^reference day 13; ^d^reference day 17.

To investigate the culture of neonatal cardiomyocytes as an *in vitro* model of cardiac cell aging, distinct biomarkers of cellular senescence were monitored (Figure 2). Herein, Ki-67 and PCNA as markers of proliferation showed a tendentially reduced mRNA expression towards day 9 and a subsequent increase in expression levels to the end of cultivation at day 21 (Figures 2(a) and 2(b)). In contrast, cell cycle inhibitors constantly increased during cardiomyocyte culture with a 33.9 ± 1.1-fold increase in p16 mRNA expression and a 6.8 ± 1.4-fold increase in p53 protein levels at day 21 compared to day 6, respectively (Figures 2(c) and 2(d)). It is noteworthy that both p16 mRNA and p53 protein showed a pronounced gain of expression in between days 9 and 13. Furthermore, nuclear signals of p21 in ACNT1-positive cardiomyocytes increased from day 6 to day 9 by a factor of 1.5 ± 0.3 but showed no further nuclear assimilation towards day 13 (Figure 2(e)). In the measured spectrum, a continuous increase in autofluorescence of contracting cardiomyocytes was determined and fluorescent signal reached a 2.5 ± 0.4-fold increase at day 21 post primary cell isolation (Figure 2(f)). The percentage of SA-*β*-Gal-positive cardiomyocytes cumulated in an exponential manner, whereupon at day 21 a plateau of 83.4 ± 5.2% visually stained heart muscle cells was determined (Figure 2(g)).

**Figure 3:**
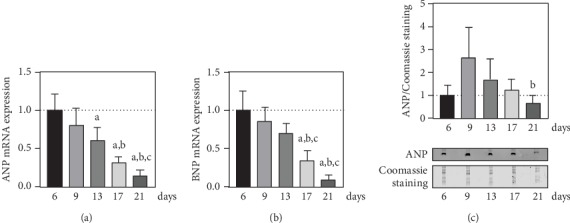
Expression of cardiac hypertrophic biomarkers in cultured neonatal cardiomyocytes. Quantitative assessment of mRNA expression for (a) ANP and (b) BNP was performed using qPCR analyses (*n* = 4 mice). (c) Secretory ANP was detected via immunoblot analyses of culture media collected at indicated time points and normalized to total protein measured by Coomassie staining (*n* = 4 mice). Pictured are representative scans of the ANP immunoblot and corresponding Coomassie staining. Data are presented as mean values ± SD. Statistical significance was assessed by one-way ANOVA (*p* < 0.05); ^a^reference day 6; ^b^reference day 9; ^c^reference day 13.

The expression profiles of ANP and BNP were used to characterize the temporal hypertrophic stress of cultured neonatal cardiomyocytes (Figure 3). For the relative mRNA expression of ANP and BNP, a distinct decrease with ongoing time in culture was observed (Figures 3(a) and 3(b)). To further evaluate these results, secretory ANP was detected in the corresponding medium supernatants of cardiomyocyte cultures (Figure 3(c)). Compared to day 6, at day 9, a 2.7 ± 1.3-fold induction of secreted ANP was determined, followed by a constant reduction towards day 21.

**Figure 4:**
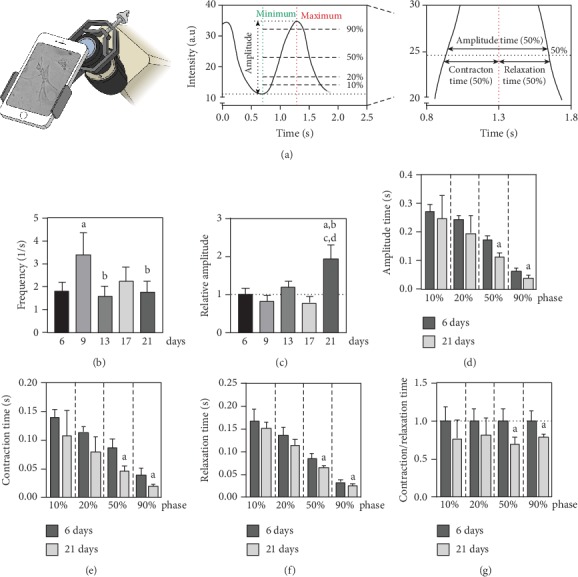
Contractility of neonatal cardiomyocytes during cultivation. Time-dependent changes in autonomous contractile behavior of murine cardiomyocytes were determined using the analytical software tool *Myocyter* (*n* = 4 mice). (a) Spontaneous contractions were recorded on a commercially available smartphone connected to the ocular of a microscope via a camera adapter. During analysis, *Myocyter* recognizes cardiomyocyte movement and calculates changes in pixel intensity. Chronologically, contractions translate to positive going transients with an arbitrary unit (a.u.). Using a dynamically determined threshold to appoint the minimum and maximum for each contraction, transients are descriptively characterized on the overall amplitude of contraction and the time spent during phases 10%, 20%, 50%, and 90% of the peak. Changes in (b) frequency and (c) relative amplitude are shown for the course of cultivation. Differences in (d) amplitude time, (e) contraction time, (f) relaxation time, and (g) the ratio of contraction per relaxation time are compared between days 6 and 21 of cultivation. Data are presented as mean values ± SD. Statistical significance was assessed by one-way ANOVA or unpaired Student's *t*-test (*p* < 0.05). ^a^Reference day 6; ^b^reference day 9; ^c^reference day 13; ^d^reference day 17.

As illustrated in Figure 4, neonatal cardiomyocytes showed distinct changes of contractile behavior in the course of cultivation. The contraction frequency significantly accelerated from 1.8 ± 0.4 Hz (1/s) at day 6 to 3.4 ± 0.9 Hz (1/s) at day 9 before decelerating again to a consistent pace on the following days 13, 17, and 21 (Figure 4(b)). The relative amplitude remained unchanged between days 6 and 17, but increased at day 21 by a factor of 1.9 ± 0.4 (Figure 4(c)). To further analyze distinct characteristics of contractility, *Myocyter* delineates the time needed for different phases of the ongoing amplitude, continuatively separated for contraction and relaxation (Figure 4(a), middle and right panels). By comparing the contractile behavior for days 6 and 21, there were no changes observed in the early phases 10% or 20%, neither in time spent during the overall amplitude (Figure 4(d)), contraction (Figure 4(e)), nor relaxation (Figure 4(f)). However, all three parameters elucidated a significant constraint of time spent during the later phases 50% and 90% of contraction peaks. The ratio of contraction per relaxation time decreased for the later phases, and therefore, a relative shift towards relaxation was shown (Figure 4(g)).

**Figure 5:**
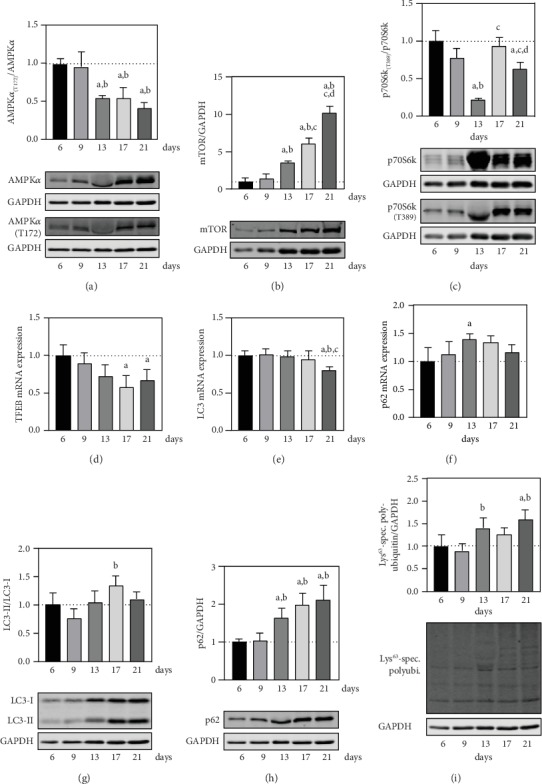
Changes in autophagy for the culture of neonatal cardiomyocytes. To assess autophagy in the culture of murine, neonatal cardiomyocytes, upstream regulators and central constituents were determined using immunoblot and qPCR analyses (*n* = 4 mice). (a) AMPK activity was measured by detecting the subunit *α* and a comparison of the basal state to its phosphorylation at Thr^172^. For the determination of mTOR activity, (b) catalytic unit mTOR and (c) the target protein p70S6k in relation to its mTOR-dependent phosphorylation at Thr^389^ were quantified. Further analyzed were time-dependent changes in relative mRNA expression of (d) TFEB, (e) LC3, and (f) p62. To measure autophagic efficiency, protein levels of (g) LC3-I in relation to LC3-II, (h) p62, and (i) Lys^63^-specific polyubiquitin were determined. Detected proteins were normalized to GAPDH as internal control, and representative immunoblots are shown. Data are presented as mean values ± SD. Statistical significance was assessed by one-way ANOVA (*p* < 0.05); ^a^reference day 6; ^b^reference day 9; ^c^reference day 13; ^d^reference day 17.

To analyze the ALS in the culture of neonatal cardiomyocytes, the major regulators AMPK and mTOR as well as key constituents of the autophagic process were investigated (Figure 5). As positive regulator of autophagy, AMPK activity itself is regulated by upstream signaling cascades and dependent on the phosphorylation of its catalytic subunit *α* at Thr^172^ [47]. For the ratio of Thr^172^ phosphorylated per basal protein, a stepwise regulation with a reduction in between days 9 and 13 was shown (Figure 5(a)). Towards the end of cultivation, no further change in phosphorylation rate was detected and the overall decrease at day 21 compared to day 6 was 0.4 ± 0.1-fold. On the other hand, with a 10.1 ± 1.0-fold induction of protein levels at day 21, mTOR showed a steady increase in the course of cultivation (Figure 5(b)). For its substrate p70S6k a significant decrease in the ratio of activating, mTOR-dependent phosphorylation at Thr^389^ per basal protein between days 9 and 13, with a following rapid increase towards day 17 was demonstrated (Figure 5(c)).

As an early transcriptional factor in ALS regulation, TFEB showed a steadily decreasing trend towards days 17 and 21 with an overall 0.7 ± 0.1-fold reduction at day 21 (Figure 5(d)). For TFEB target genes LC3 and p62, only minor changes in mRNA expression levels were measured. The relative mRNA expression of LC3 was unchanged from day 6 to day 17 and decreased on day 21 by a factor of 0.8 ± 0.1 (Figure 5(e)). Expression levels of p62 showed a 1.4 ± 0.1-fold induction towards day 13 with a subsequent downward trend until day 21 post primary cell isolation (Figure 5(f)). For the proportion of lipidated LC3-II per unconjugated LC3-I, a reduction by trend between days 6 and 9, a following induction until day 17, and a final minor regression on day 21 were determined (Figure 5(g)). With no change in protein levels from day 6 to day 9 and a subsequent accumulation on days 13, 17, and 21, the overall profiles of p62 and Lys^63^-linkage specific polyubiquitination reflected the same time-dependent tendencies (Figures 5(h) and 5(i)). The total induction of p62 protein and Lys^63^-linkage specific polyubiquitination on day 21 compared to day 6 was 2.1 ± 0.4 and 1.6 ± 0.2-fold, respectively.

To verify analyses on the occurring autophagic flux, experiments with a ConA treatment in comparison to basal culture conditions were performed (Suppl. Figures [Supplementary-material supplementary-material-1] and [Supplementary-material supplementary-material-1]).

## 4. Discussion

Being associated with a decline of intrinsic physiological function, aging must be delineated depending on biomarkers independently of chronological age. Despite being a heterogeneous, stimuli- and cell type-specific phenomenon, senescent cells accumulate in distinct organs of humans, primates, and rodents during aging and age-associated pathologies [48, 49]. Recent research reveals an increase of senescence-associated biomarkers in the heart with age [50].

According to its definition, cellular senescence is negatively associated with proliferation rate. An efficient regenerative potential of murine cardiomyocytes seems to be restricted to a neonatal period of 7 days after birth [51]. Following a phase of endoreplication, in mice 85-90% of cardiac myocytes reach a seemingly postmitotic state at the age of 21 days [52]. Thus, proliferation rate of isolated heart muscle cells from neonatal mice should decrease during 21 days in culture. Nevertheless, in the herein described cultivation of neonatal cardiomyocytes, expression of proliferation markers Ki-67 and PCNA showed a steady increase after 9 days post isolation (Figures 2(a) and 2(b)). Most likely and as evidenced by the described growth of ACTN1-negative cells during microscopic analyses, the suggested increase in proliferative activity is attributable to noncardiomyocyte populations. Thus, the observation of the proliferation markers could indicate the expression levels of different cell populations which are superimposed in a proportionally inverse manner. While the noncardiomyocytes as proliferating cells are constantly increasing, the percentage of heart muscle cells is continuously decreasing. The murine adult myocardium shows a cellular distribution of approximately 56% cardiomyocytes, 27% fibroblasts, 10% vascular myocytes, and 7% endothelial cells [53]. Furthermore, during neonatal development, Banerjee et al. described a cumulative increase of cardiac fibroblasts of 51% between postnatal days 1 and 15 in the murine heart [53]. Thus, for further interpretation of presented results, it is necessary to differentiate between cardiomyocyte-specific investigations and the culture as heterogeneous cell system.

In principle, molecular activation of cellular senescence is tied to two signaling pathways for the inhibition of cell cycle progression and proliferation. These include activation of p16 as an inhibitor of CDK4/6 and stabilization of p53, leading to the upregulation of p21 as an inhibitor of CDK2 [48, 49]. For the 21-day culture of neonatal cardiomyocytes, a consistent increase of p16 at mRNA level and of p53 on protein level was observed over time (Figures 2(c) and 2(d)). Regarding the proliferative activity of different cell populations, despite being determined for total lysates, the induction of cell cycle inhibitors should be attributable to cardiomyocytes. Supportingly, immunofluorescence staining showed an increase in p21 nuclear assimilation in ACTN1-positive cells between days 6 and 9, even though no further increase was observed for the following time point at 13 days post primary cell isolation (Figure 2(e)). All in all, this supports the assumption of a time-dependent induction of both axes of cell cycle inhibition for the culture system of neonatal cardiomyocytes.

Further microscopic analyses revealed the onset of an aging-associated senescent phenotype for the cultured cardiac myocytes in particular. Within 21 days under culture conditions, a significant increase in autofluorescence of contracting cardiomyocytes, indicating the accumulation of lipofuscin-like protein aggregates, and the percentage of SA-*β*-Gal-positive heart muscle cells was observed (Figures 2(f) and 2(g)). Accumulation of oxidized and cross-linked protein aggregates, such as lipofuscin, is regarded as a characteristic of postmitotic aging [54, 55]. Another aspect is the 1.8-fold increase in cellular size of ACTN1-positive cardiomyocytes between days 6 and 13 post isolation (Figure 1). Recent research brought light into the inverse correlation of excessive cell growth and proliferative decline up to cellular senescence [56], a phenomenon already described empirically by Hayflick and Moorhead [57]. The postulated molecular mechanism is a regulatory imbalance, whereby a progressive dilution of the cytosol leads to a limitation of the DNA [56].

Overall, our results correspond with Wang et al., who have already described the culture of neonatal cardiomyocytes derived from C57Bl/6 mice over 28 days as a model system of cardiac senescence [40]. The herein presented results supplement the literature by the data of p16, p21 and autofluorescence in cultured neonatal cardiomyocytes and clarify the temporal course of the commonly used markers of cellular senescence over 21 days. Nevertheless, there remains the question of why such markers of biological aging accumulate in chronologically young cells. Model systems *in vitro* distinguish between chronic, replicative and acute stress-induced premature senescence (SIPS) [58]. With an increase in p16, p53, p21, and SA-*β*-Gal activity, our results are in agreement with a doxorubicin-induced SIPS in rat neonatal cardiomyocytes [59]. Puente et al. proposed an oxygen-rich environment after birth as key factor of cardiomyocyte cell cycle withdrawal during postnatal mammalian development through oxidatively induced DNA damage response [60]. We conclude that oxygen-rich conditions during culture could contribute to the entry of the age-related, senescent phenotype in cultured neonatal cardiomyocytes.

There are reports of model systems of cellular senescence with a cell type-exclusive phenotype [58]. A mutual relationship between cardiac senescence and hypertrophic remodulation of the heart was found in a murine model of pathological cardiac hypertrophy [61]. The primary cell isolation itself may be regarded as an induced cardiac tissue injury, whereupon the response of postmitotic cardiomyocytes is limited to cellular hypertrophy. Despite the increase in cellular size, mRNA levels of ANP and BNP as markers of hypertrophy decreased during cultivation with supposedly high expression levels in the beginning of cardiomyocyte culture (Figures 3(a) and 3(b)). If the noncardiomyocyte population continues to increase over time, the total lysates show a steady dilution of the mRNA of a cardiomyocyte-specific gene product. However, in the medium supernatant, the highest level of ANP protein secretion was found on day 9 and again decreased up to day 21 of cardiomyocyte culture (Figure 3(c)). This corresponded to the mRNA expression, offset by one measuring point, and thus verified the mRNA data. In conclusion, ANP and BNP expression profiles mark a regenerative, hypertrophic stress with the highest response on day 9. As an *in vitro* system, cultured neonatal cardiomyocytes are not being exposed to a constitutive hemodynamic load. Hence, they can reach a compensatory state of cellular hypertrophy, which negates the necessity of further expression of an adaptive hormonal response.

Subsequently, we were also interested in the functional capacity of the culture system. The main stimulus for the secretion of ANP is the mechanical stretching of cardiomyocytes and takes place via the activation of strain-sensitive ion channels as effective mechanosensors [62]. A murine model system of atrial tachycardia and isolated cardiomyocytes from neonatal rats showed a direct dependence of ANP secretion on contraction frequency [63, 64]. Our measurements of the contraction frequency of cultured neonatal cardiomyocytes showed a clear temporal agreement with ANP secretion (Figure 4(b)). In the period between days 6 and 9, the largest hypertrophic stimulation, the contraction frequency increased by a factor of 1.9 and decelerated to a uniform mean by days 13 to 21. It is difficult to define a normal state for the artificial system. Tiemann et al. determined a heart rate of 6.6 Hz (1/s) for C57Bl/6 mice on day 21 after birth, which rose to 9.2 Hz (1/s) by day 50 and then remained constant [65]. The contraction frequency of isolated cardiomyocytes from neonatal rats has already been extensively characterized. Here, a frequency of 1.5-2.5 Hz (1/s) was determined after 1 to 5 days in culture [66, 67], with a clear dependence on temperature, pH value, and coating medium [68, 69], which complicates overall comparability in the literature. For the present culture of murine neonatal heart muscle cells, a stress-dependent increase of contraction frequency can be assumed for day 9, which then normalized to day 13 and remained constant until day 21.

In cultured neonatal cardiomyocytes, the relative contraction amplitude showed no change between days 6 and 17, but significantly increased on day 21 (Figure 4(c)). Thus, despite the acute hypertrophy and age-related cellular senescence of cardiac myocytes, the extent of cell shortening remained mostly constant and even increased at the end of cultivation. On a translational level, this could mean a compensatory maintenance of overall contractility. However, it should be discussed that the image evaluation of the amplitudes used, *i.e*., the calculation of pixel changes as motions, does not represent an absolute value of cell shortening. Thus, the amplitudes depend on the transparency of a cardiomyocyte and the area ratio of the cell to the overall image. An increase in the granularity of the cells over time would lead to an overestimation of the amplitudes compared to the actual state. Therefore, it is essential to further characterize the contractile behavior of cardiomyocytes with absolute parameters.

For the following analyses of time-dependent changes in the percental phases of contraction, we compared day 6, after equilibration of the cardiomyocytes in the allegedly young state, and day 21, after termination of the acute hypertrophic stimulation and resulting accumulation of senescent biomarkers. The trends illustrated correspond to the temporal changes over the full course of 21-day cultivation (data not shown). The observed decrease in time spent during the phases 50% and 90% for amplitude (Figure 4(d)), contraction (Figure 4(e)), and relaxation time (Figure 4(f)) implies a narrowing and timely constraint in the late contraction peak. Due to the increase of relative cell shortening on day 21, a further time-dependent acceleration of contraction and relaxation speed can be concluded. In addition, the declined ratio of contraction per relaxation time for the later phases 50% and 90% after 21 days suggests a time shift of the late amplitude towards a relative prolongation of relaxation (Figure 4(g)).

Age-dependent changes in contractility of cardiomyocytes from adult mice have already been extensively studied. Rising evidence suggests an age-related reduction in amplitude dimension, prolonged time to peak contraction, and a slowed relaxation [70, 71]. These observations coincide with different analyses on intact hearts *in vivo* and in the hemodynamic context, which showed smaller and slower contractions depending on age [70, 71]. This contradicts the results of our 21-day culture of neonatal cardiomyocytes. Despite the time-dependent, stress-induced impairment of the late contraction phases, the overall contraction capacity, as seen by the relative amplitude, was compensated for. We conclude that the herein described model system represents an acute stress situation, which, on a functional level, can only to a limited extent be transferred to the chronic hemodynamic stress of the aging heart. Still, by choosing distinct time points, the culture of neonatal heart muscle cells may represent contractile changes during cardiac hypertrophy.

After establishing the culture of neonatal cardiomyocytes as a partial model of cardiac aging, the ALS was characterized during 21 days of cultivation. The following results were conducted for the total lysates and thus represent the complete, heterogeneous culture system.

Being central regulators of ALS, the mutual relationship between AMPK and mTOR must be considered in parallel. The rate of activating phosphorylation of subunit AMPK*α* indicates a reduction of proautophagic AMPK activity between days 9 and 13, which remains reduced until day 21 (Figure 5(a)). In an inverse correlation, accumulation of the catalytic unit mTOR on protein level indicates a constant increase in antiautophagic mTOR signaling between days 13 and 21 for the culture system (Figure 5(b)). The mTOR-dependent phosphorylation profile of its substrate p70S6k initially suggests the lowest mTOR activity on day 13 (Figure 5(c)). However, this might be due to the high protein level of basal p70S6k at this point in time and could therefore be indicative of an early response to increased protein synthesis as signaled by low AMPK and high mTOR activity. In total, between days 9 and 13, an alleged regulatory switch between an initially high AMPK and subsequent increased mTOR signaling can be demonstrated. To bring the initial observations further into a physiological context and in relation to ALS, the investigations on the course of autophagy during cultivation must first be characterized more precisely.

The decrease in mRNA expression of TFEB during culture indicates a time-dependent reduction of the central transcription factor of autophagy (Figure 5(d)). The expression profile of TFEB is confirmed by a delayed reduction in the mRNA levels of LC3, a target gene of TFEB, between days 17 and 21 (Figure 5(e)). However, observed changes of LC3, p62, and Lys^63^-linkage specific polyubiquitinated substrates on protein level allow no clear interpretation of autophagic activity, if considered in the basal state alone. With a mostly unchanged mRNA expression of LC3 and no detectable change in LC3-I during culture (Suppl. [Supplementary-material supplementary-material-1]), differences in the ratio of lipidated LC3-II per unconjugated LC3-I are mostly attributable to detection levels of LC3-II (Suppl. [Supplementary-material supplementary-material-1]). The expression of p62 on mRNA level remained largely unchanged, which does not indicate major differences in its transcriptional regulation (Figure 5(f)). Increasing protein LC3-II (Figure 5(g)), p62 (Figure 5(h)), and Lys^63^-polyubiquitinated substrates (Figure 5(i)) following day 9 could be indicative of a rise in autophagy flux to cope with increasing autophagic substrates. However, neither upstream AMPK and mTOR signaling nor transcriptional levels of TFEB, LC3, and p62 support a time-dependent activation of the ALS. An increase in detectable LC3-II, p62, and autophagic substrates could also point to a malfunctioning fusion of autophagosomes with lysosomes, an impaired degradation of cargo in the autolysosome or both, resulting in an accumulation of these proteins in the cell. Considering all measured parameters, we assume a maximum activity of the ALS on day 9, which then decreases until day 17. Nevertheless, a more valid conclusion can only be drawn by comparing the respective detection levels of autophagic proteins in the presence and absence of ALS inhibitors, such a ConA.

Therefore, we performed comparative analyses of autophagic proteins LC3 and p62 under basal conditions in relation to a ConA-induced blockade of lysosomal degradation. By specifically monitoring the transition point between days 6, 9, and 13, where we assumed the beginning of a restricted autophagic activity, we initially compared the ratio of LC3-II/LC3-I in basal state and ConA-induced conditions (Suppl. [Supplementary-material supplementary-material-1]). As the relative difference between control and ConA treatment increases from day 6 to day 9, an increasing autophagy flux can be assumed which does not change towards day 13. Additionally, detection levels of p62 confirm an increase in autophagy between days 6 and 9 as the ConA-induced accumulation of the protein increases significantly (Suppl. [Supplementary-material supplementary-material-1]. Comparison of p62 with and without lysosomal inhibition demonstrates the highest turnover rate of the protein on day 9. Moreover, autophagy flux seems to become impaired from day 13 on, as the ConA-induced state does not differ from the basal condition, suggesting a reduced protein turnover, already indicated by an increasing trend of p62 under basal conditions.

Summarizing, for the 21-day culture of neonatal cardiomyocytes, we could detect a possible rise in autophagy flux up to day 9. However, between days 9 and 13, a regulatory switch in AMPK and mTOR signaling suggests a continuative inhibition of autophagy. Furthermore, autophagic adaptor proteins LC3-II and p62 as well as Lys^63^-polyubiquitinated autophagy substrates increase between days 13 and 21, indicating an impairment of the ALS in the aging cells.

Thus, our investigations in the *in vitro* culture system are comparable with observations on an age-dependent decrease of autophagy in heart tissue of C57Bl/6J mice [26] and general, species-spanning analyses of the ALS during aging *in vivo* [72]. Lysosomal accumulation of oxidized and cross-linked protein aggregates, such as lipofuscin, is described as a central factor of an age-related reduced capacity of the ALS [54, 73, 74]. In the course of the 21-day cultivation of murine neonatal cardiomyocytes, an oxygen-rich environment and acute hypertrophic stimulus appear to imbalance cellular homeostasis. This could decisively contribute to the development of the described SIPS phenotype. For the present culture system, an association between increasing markers of senescence-associated cardiac aging and an impaired ALS was shown.

## 5. Conclusions

Within the present study, we defined a 21-day culture of cardiomyocytes derived from neonatal C57Bl/6J mice as a model system of cardiac aging. By classifying the time-dependent changes in cardiac-specific hypertrophy, contractility, and autophagy, we described a dysregulation of cellular homeostasis which we further discussed as cause of premature entry of cardiac myocytes into cellular senescence. The herein established model may complement and possibly reduce animal studies which are conducted to illuminate the molecular mechanisms of the aging heart.

## Data Availability

The experimental data used to support the findings of this study are available from the corresponding author upon request.

## Abbreviations

ACTN1: *α*-Actinin
AMPK: AMP-activated protein kinase
ANP: Atrial natriuretic peptide
ALS: Autophagy-lysosomal system
BNP: Brain natriuretic peptide
ConA: Concanamycin A
GAPDH: Glyceraldehyde-3-phosphate dehydrogenase
LC3: Microtubule-associated protein 1 light chain 3
mTOR: Mechanistic target of rapamycin kinase
PCNA: Proliferating cell nuclear antigen
p62: Sequestosome 1
p70S6k: p70 S6 kinase
qPCR: Real-time PCR
SA-*β*-Gal: Senescence-associated *β*-galactosidase
SIPS: Stress-induced premature senescence
TFEB: Transcription factor EB.

## Supplementary Materials

Supplementary Materials Suppl. Figure 1: Further analyses on autophagy in the 21-day culture of neonatal cardiomyocytes.

## References

[1] López-Otín C., Blasco M. A., Partridge L., Serrano M., Kroemer G. (2013). The hallmarks of aging. *Cell*.

[2] World Health Organization (WHO) (2016). *Global Health Estimates 2016: Deaths by Cause, Age, Sex, by Country and by Region, 2000-2016*.

[3] World Health Organization (WHO) (2018). *World Health Statistics 2018: Monitoring Health for the SDGs, Sustainable Development Goals*.

[4] McAloon C. J., Osman F., Glennon P., Lim P. B., Hayat S. A., Papageorgiou N. (2016). Global epidemiology and incidence of cardiovascular disease. *Cardiovascular Disease - Genetic Susceptibility, Enviromental Factors and their Interaction*.

[5] Obas V., Vasan R. S. (2018). The aging heart. *Clinical Science*.

[6] Nakou E. S., Parthenakis F. I., Kallergis E. M., Marketou M. E., Nakos K. S., Vardas P. E. (2016). Healthy aging and myocardium: a complicated process with various effects in cardiac structure and physiology. *International Journal of Cardiology*.

[7] Chiba A., Watanabe-Takano H., Miyazaki T., Mochizuki N. (2018). Cardiomyokines from the heart. *Cellular and Molecular Life Sciences*.

[8] Shioi T., Inuzuka Y. (2012). Aging as a substrate of heart failure. *Journal of Cardiology*.

[9] Bernhard D., Laufer G. (2008). The aging cardiomyocyte: a mini-review. *Gerontology*.

[10] Bergmann O., Zdunek S., Felker A. (2015). Dynamics of cell generation and turnover in the human heart. *Cell*.

[11] Walsh S., Pontén A., Fleischmann B. K., Jovinge S. (2010). Cardiomyocyte cell cycle control and growth estimation in vivo - an analysis based on cardiomyocyte nuclei. *Cardiovascular Research*.

[12] Shirakabe A., Ikeda Y., Sciarretta S., Zablocki D. K., Sadoshima J. (2016). Aging and autophagy in the heart. *Circulation Research*.

[13] Sciarretta S., Maejima Y., Zablocki D., Sadoshima J. (2018). The role of autophagy in the heart. *Annual Review of Physiology*.

[14] Korovila I., Hugo M., Castro J. P. (2017). Proteostasis, oxidative stress and aging. *Redox Biology*.

[15] Dikic I., Elazar Z. (2018). Mechanism and medical implications of mammalian autophagy. *Nature Reviews Molecular Cell Biology*.

[16] Ravikumar B., Sarkar S., Davies J. E. (2010). Regulation of mammalian autophagy in physiology and pathophysiology. *Physiological Reviews*.

[17] Parzych K. R., Klionsky D. J. (2014). An overview of autophagy: morphology, mechanism, and regulation. *Antioxidants & Redox Signaling*.

[18] König J., Grune T., Ott C. (2019). Assessing autophagy in murine skeletal muscle: current findings to modulate and quantify the autophagic flux. *Current Opinion in Clinical Nutrition and Metabolic Care*.

[19] Pankiv S., Clausen T. H., Lamark T. (2007). p62/SQSTM1 binds directly to Atg8/LC3 to facilitate degradation of ubiquitinated protein aggregates by autophagy. *Journal of Biological Chemistry*.

[20] Mizushima N., Yoshimori T. (2007). How to interpret LC3 immunoblotting. *Autophagy*.

[21] Gómez-Sánchez R., Pizarro-Estrella E., Yakhine-Diop S. M. (2015). Routine Western blot to check autophagic flux: cautions and recommendations. *Analytical Biochemistry*.

[22] Klionsky D. J., Abdelmohsen K., Abe A. (2016). Guidelines for the use and interpretation of assays for monitoring autophagy (3rd edition). *Autophagy*.

[23] Settembre C., Di Malta C., Polito V. A. (2011). TFEB links autophagy to lysosomal biogenesis. *Science*.

[24] Rabanal-Ruiz Y., Otten E. G., Korolchuk V. I. (2017). mTORC1 as the main gateway to autophagy. *Essays in Biochemistry*.

[25] Nakai A., Yamaguchi O., Takeda T. (2007). The role of autophagy in cardiomyocytes in the basal state and in response to hemodynamic stress. *Nature Medicine*.

[26] Taneike M., Yamaguchi O., Nakai A. (2010). Inhibition of autophagy in the heart induces age-related cardiomyopathy. *Autophagy*.

[27] Gonzalez A. A., Kumar R., Mulligan J. D., Davis A. J., Saupe K. W. (2004). Effects of aging on cardiac and skeletal muscle AMPK activity: basal activity, allosteric activation, and response to in vivo hypoxemia in mice. *American Journal of Physiology-Regulatory, Integrative and Comparative Physiology*.

[28] Yang Z., Ming X. F. (2012). mTOR signalling: the molecular interface connecting metabolic stress, aging and cardiovascular diseases. *Obesity Reviews*.

[29] Hoes M. F., Bomer N., van der Meer P. (2019). Concise review: the current state of human in vitro cardiac disease modeling: a focus on gene editing and tissue engineering. *Stem Cells Translational Medicine*.

[30] Louch W. E., Sheehan K. A., Wolska B. M. (2011). Methods in cardiomyocyte isolation, culture, and gene transfer. *Journal of Molecular and Cellular Cardiology*.

[31] Boheler K. R., Czyz J., Tweedie D., Yang H. T., Anisimov S. V., Wobus A. M. (2002). Differentiation of pluripotent embryonic stem cells into cardiomyocytes. *Circulation Research*.

[32] Takahashi K., Tanabe K., Ohnuki M. (2007). Induction of pluripotent stem cells from adult human fibroblasts by defined factors. *Cell*.

[33] Claycomb W. C., Lanson N. A., Stallworth B. S. (1998). HL-1 cells: a cardiac muscle cell line that contracts and retains phenotypic characteristics of the adult cardiomyocyte. *Proceedings of the National Academy of Sciences of the United States of America*.

[34] Davidson M. M., Nesti C., Palenzuela L. (2005). Novel cell lines derived from adult human ventricular cardiomyocytes. *Journal of Molecular and Cellular Cardiology*.

[35] Parameswaran S., Kumar S., Verma R. S., Sharma R. K. (2013). Cardiomyocyte culture - an update on the in vitro cardiovascular model and future challenges. *Canadian Journal of Physiology and Pharmacology*.

[36] Fu J. J., Gao H., Pi R. B., Liu P. Q. (2005). An optimized protocol for culture of cardiomyocyte from neonatal rat. *Cytotechnology*.

[37] Liu X., Deng Y., Xu Y., Jin W., Li H. (2018). MicroRNA-223 protects neonatal rat cardiomyocytes and H9c2 cells from hypoxia- induced apoptosis and excessive autophagy via the Akt/mTOR pathway by targeting PARP-1. *Journal of Molecular and Cellular Cardiology*.

[38] Böckmann I., Lischka J., Richter B. (2019). FGF23-mediated activation of local RAAS promotes cardiac hypertrophy and fibrosis. *International Journal of Molecular Sciences*.

[39] Ghasemi Tahrir F., Gupta M., Myers V. (2019). Role of Bcl2-associated athanogene 3 in turnover of gap junction protein, connexin 43, in neonatal cardiomyocytes. *Scientific Reports*.

[40] Wang Z., Rong X., Luo B. (2016). A natural model of mouse cardiac myocyte senescence. *Journal of Cardiovascular Translational Research*.

[41] Grune T., Ott C., Häseli S., Höhn A., Jung T. (2019). The “MYOCYTER” - convert cellular and cardiac contractions into numbers with ImageJ. *Scientific Reports*.

[42] Croce A. C., Bottiroli G., Pellicciari C., Biggiogera M. (2017). Autofluorescence spectroscopy for monitoring metabolism in animal cells and tissues. *Histochemistry of Single Molecules*.

[43] Jung T., Bader N., Grune T. (2007). Lipofuscin: formation, distribution, and metabolic consequences. *Annals of the New York Academy of Sciences*.

[44] Jung T., Höhn A., Grune T., Armstrong D. (2010). Lipofuscin: detection and quantification by microscopic techniques. *Advanced Protocols in Oxidative Stress II*.

[45] Debacq-Chainiaux F., Erusalimsky J. D., Campisi J., Toussaint O. (2009). Protocols to detect senescence-associated beta-galactosidase (SA-*β*gal) activity, a biomarker of senescent cells in culture and *in vivo*. *Nature Protocols*.

[46] Vandesompele J., De Preter K., Pattyn F. (2002). Accurate normalization of real-time quantitative RT-PCR data by geometric averaging of multiple internal control genes. *Genome Biology*.

[47] Qi D., Young L. H. (2015). AMPK: energy sensor and survival mechanism in the ischemic heart. *Trends in Endocrinology & Metabolism*.

[48] van Deursen J. M. (2014). The role of senescent cells in ageing. *Nature*.

[49] Childs B. G., Durik M., Baker D. J., van Deursen J. M. (2015). Cellular senescence in aging and age-related disease: from mechanisms to therapy. *Nature Medicine*.

[50] Anderson R., Richardson G. D., Passos J. F. (2018). Mechanisms driving the ageing heart. *Experimental Gerontology*.

[51] Porrello E. R., Mahmoud A. I., Simpson E. (2011). Transient regenerative potential of the neonatal mouse heart. *Science*.

[52] Ahuja P., Sdek P., MacLellan W. R. (2007). Cardiac myocyte cell cycle control in development, disease, and regeneration. *Physiological Reviews*.

[53] Banerjee I., Fuseler J. W., Price R. L., Borg T. K., Baudino T. A. (2007). Determination of cell types and numbers during cardiac development in the neonatal and adult rat and mouse. *American Journal of Physiology-Heart and Circulatory Physiology*.

[54] Reeg S., Grune T. (2015). Protein oxidation in aging: does it play a role in aging progression?. *Antioxidants & Redox Signaling*.

[55] Höhn A., Weber D., Jung T. (2017). Happily (n)ever after: aging in the context of oxidative stress, proteostasis loss and cellular senescence. *Redox Biology*.

[56] Neurohr G. E., Terry R. L., Lengefeld J. (2019). Excessive cell growth causes cytoplasm dilution and contributes to senescence. *Cell*.

[57] Hayflick L., Moorhead P. S. (1961). The serial cultivation of human diploid cell strains. *Experimental Cell Research*.

[58] Sikora E., Bielak-Zmijewska A., Mosieniak G. (2014). Cellular senescence in ageing, age-related disease and longevity. *Current Vascular Pharmacology*.

[59] Maejima Y., Adachi S., Ito H., Hirao K., Isobe M. (2008). Induction of premature senescence in cardiomyocytes by doxorubicin as a novel mechanism of myocardial damage. *Aging Cell*.

[60] Puente B. N., Kimura W., Muralidhar S. A. (2014). The oxygen-rich postnatal environment induces cardiomyocyte cell-cycle arrest through DNA damage response. *Cell*.

[61] Sun R., Zhu B., Xiong K. (2017). Senescence as a novel mechanism involved in *β*-adrenergic receptor mediated cardiac hypertrophy. *PLoS One*.

[62] Dietz J. R. (2005). Mechanisms of atrial natriuretic peptide secretion from the atrium. *Cardiovascular Research*.

[63] Church D. J., Rebsamen M. C., Morabito D., van Der Bent V., Vallotton M. B., Lang U. (2000). Role of cell contractions in cAMP-induced cardiomyocyte atrial natriuretic peptide release. *American Journal of Physiology-Heart and Circulatory Physiology*.

[64] Schiebinger R. J., Linden J. (1986). Effect of atrial contraction frequency on atrial natriuretic peptide secretion. *American Journal of Physiology-Heart and Circulatory Physiology*.

[65] Tiemann K., Weyer D., Djoufack P. C. (2003). Increasing myocardial contraction and blood pressure in C57BL/6 mice during early postnatal development. *American Journal of Physiology-Heart and Circulatory Physiology*.

[66] Rogers T. B., Gaa S. T., Allen I. S. (1986). Identification and characterization of functional angiotensin II receptors on cultured heart myocytes. *The Journal of Pharmacology and Experimental Therapeutics*.

[67] Weisensee D., Seeger T., Bittner A., Bereiter-Hahn J., Schoeppe W., Löw-Friedrich I. (1995). Cocultures of fetal and adult cardiomyocytes yield rhythmically beating rod shaped heart cells from adult rats. *In Vitro Cellular & Developmental Biology-Animal*.

[68] Harary I., Farley B. (1963). *In vitro* studies on single beating rat heart cells: II. Intercellular communication. *Experimental Cell Research*.

[69] Miskon A., Ehashi T., Mahara A., Uyama H., Yamaoka T. (2009). Beating behavior of primary neonatal cardiomyocytes and cardiac-differentiated P19.CL6 cells on different extracellular matrix components. *Journal of Artificial Organs*.

[70] Feridooni H. A., Kane A. E., Ayaz O. (2017). The impact of age and frailty on ventricular structure and function in C57BL/6J mice. *The Journal of Physiology*.

[71] Feridooni H. A., Dibb K. M., Howlett S. E. (2015). How cardiomyocyte excitation, calcium release and contraction become altered with age. *Journal of Molecular and Cellular Cardiology*.

[72] Cuervo A. M., Bergamini E., Brunk U. T., Dröge W., Ffrench M., Terman A. (2005). Autophagy and aging: the importance of maintaining “clean” cells. *Autophagy*.

[73] Brunk U. T., Terman A. (2002). Lipofuscin: mechanisms of age-related accumulation and influence on cell function. *Free Radical Biology and Medicine*.

[74] Höhn A., Grune T. (2013). Lipofuscin: formation, effects and role of macroautophagy. *Redox Biology*.

