# Supplementary material for: Cardiomyocyte Contractility and Autophagy in a Premature Senescence Model of Cardiac Aging
Source: Oxid Med Cell Longev. 2020 Apr 14;2020:8141307. doi: 10.1155/2020/8141307 (PMC7180990; doi:10.1155/2020/8141307)
Supplement: Supplementary Materials — Suppl. Figure 1: Further analyses on autophagy in the 21-day culture of neonatal cardiomyocytes. [file 8141307.f1.docx]

**Cardiomyocyte contractility and autophagy in a premature senescence model of cardiac aging**

Steffen Häseli, Stefanie Deubel, Tobias Jung, Tilman Grune, Christiane Ott

**Supplementary Materials**


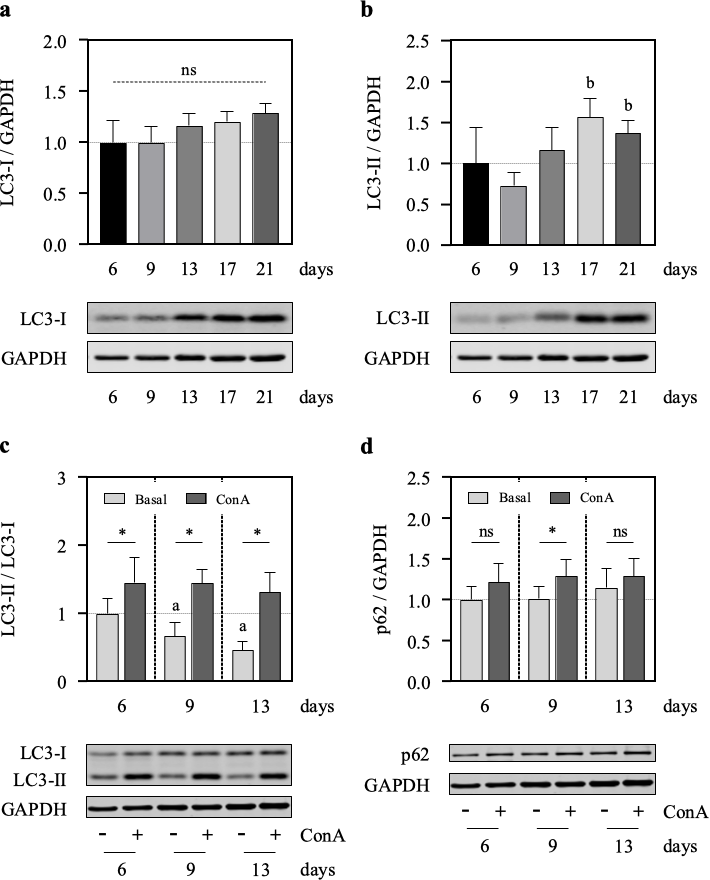


**Suppl. Figure 1. Further analyses on autophagy in the 21-day culture of neonatal cardiomyocytes.** To further clarify cellular autophagic response, neonatal cardiomyocyte immunoblot analyses (n = 4 mice) of **(a)** LC3-I and **(b)** LC3-II are provided. Additionally, cells were treated with concanamycin A (ConA) for 6 h and comparative analyses with respective controls (basal) were conducted. To assess indications on autophagic flux, immunoblot analyses (n = 7 mice) were performed to determine time-dependent changes in protein levels of **(c)** LC3-I in relation to LC3-II and **(d)** p62. Detected proteins were normalized to GAPDH as internal control and representative immunoblots are shown. Data are presented as mean values ± SD. Statistical significance was assessed by one-way ANOVA or unpaired *Student’s* t-test (p < 0.05). ^a^, reference day 6 (basal); ^b^, reference day 9; ^ns^, not significant.
